# β-carboline derivative Z86 alleviates pulmonary fibrosis via suppressing TGF-β/Smad signaling

**DOI:** 10.1007/s13659-026-00628-w

**Published:** 2026-08-28

**Authors:** Yuyang Wang, Shaohua Zhang, Rong Geng, Ruiying Wang, Ying Huang, Gang Xu, Lingmei Kong, Yan Li

**Affiliations:** 1 Key Laboratory of Medicinal Chemistry for Natural Resource, Ministry of EducationYunnan Key Laboratory of Research and Development for Natural Products, School of Pharmacy, Yunnan University, Kunming, 650500 P.R. China; 2 State Key Laboratory of Phytochemistry and Plant Resources in West China, Kunming Institute of Botany, Chinese Academy of Sciences, Kunming, 650201 People’s Republic of China

**Keywords:** Z86, Pulmonary fibrosis, TGF-β, Smad2/3, USP15

## Abstract

Idiopathic pulmonary fibrosis (IPF) is characterized by aberrant fibroblast proliferation and excessive expression and deposition of extracellular matrix (ECM) by myofibroblasts, ultimately resulting in progressive and irreversible disorder or even loss of the pulmonary function. IPF is incurable with limited therapeutic options and low survival rate, thus the discovery of the therapeutic potential agents for IPF is in urgent need. Here, we identified isopropyl 9-ethyl-1-(naphthalen-1-yl)-9H-pyrido[3,4-b] indole-3-carboxylate (Z86) as a promising bioactive agent against Bleomycin (BLM) induced pulmonary fibrotic mice with the lung abnormalities including histological alteration, accumulation of ECM, as well as markers of EMT (epithelial-mesenchymal transition) were all relieved upon Z86 treatment in C57BL/6 mice. Consistent to the activity in vivo, Z86 decreased TGF-β induced cell migration, fibrotic marker expression, and myofibroblast activation in A549 and HFL1 cells. In further mechanism of action study of Z86 against IPF, we found TGF-β (transforming growth factor β) /Smad2/3 signaling pathway was substantially attenuated in TGF-β treated A549 and HFL1 cells, with remarkably decreased protein level of Smad2/3. Further, we identified Z86 as a novel USP15 inhibitor. Correspondingly, Z86 interacted with USP15, promoted the ubiquitination and degradation of Smad2/3 and disrupted TGF-β/Smad2/3 signaling pathway. Taken together, our findings demonstrated that Z86 suppressed TGF-β induced pulmonary fibrosis in A549 and HFL cells and BLM induced pulmonary fibrotic mice through inhibiting TGF-β/Smad2/3 signaling, and provided the possibility of USP15 inhibitors as the potential drug candidates for idiopathic pulmonary fibrosis.

**Graphical Abstract:**

**Figure:**
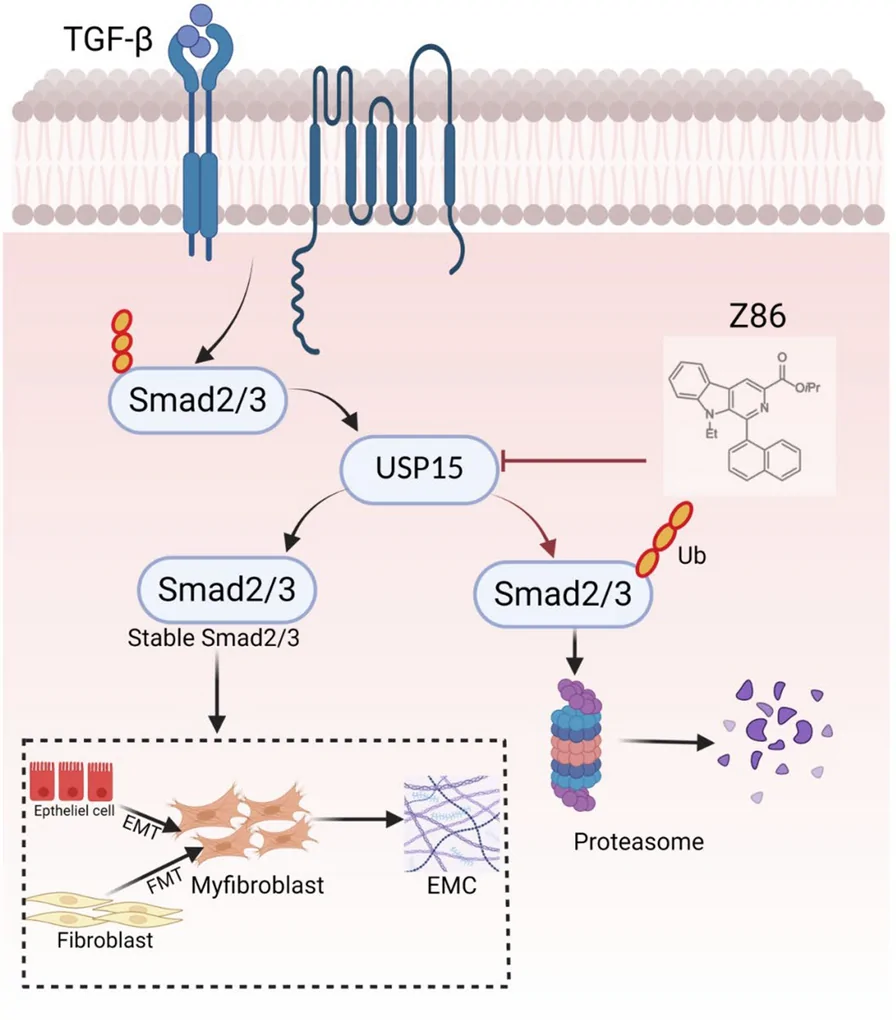

## Highlights

- Z86 suppresses BLM induced pulmonary fibrosis in mice.
- Z86 reverses TGF-β induced EMT in pulmonary fibroblast cells.
- Z86 attenuates TGF-β caused ECM deposition in pulmonary fibroblast cells.
- Z86 induces remarkable post-transcriptional downregulation of Smad2/3.
- Z86 targets USP15 to promote the degradation of Smad2/3.

## Introduction

Idiopathic pulmonary fibrosis (IPF) is an advanced and fatal lung disease with increasing incidence and prevalence. IPF is characterized by lung fibrosis, constant inflammation and destruction of lung architecture, ultimately leading to respiratory failure and death. To date, IPF is incurable, with a survival rate of 3–5 years after diagnosis [1–3]. Currently, the available therapies are the oral anti-fibrotic drugs Pirfenidone and Nintedanib, which reduce symptoms and slow down disease progression, however, with the survival or quality of life remaining unchanged. And, lots of patients who take them endure long-term experience of adverse effects and significant withdrawal symptoms. Unilateral or bilateral lung transplantation is the only treatment for IPF that can prolong life expectancy, but it cannot be widely performed due to technical and donor organ quantity limitation [4–6]. Therefore, there is an urgent need to develop novel anti-IPF drugs with more efficiency and improved safety.

The pathogenesis of IPF is not fully understood, and it is currently believed that fibrotic diseases are typically characterized by dysregulation of the injury repairing process in lung epithelial cells, with persistent secretion of multiple cell chemokines, including TGF-β, platelet-derived growth factor (PDGF), interleukin (IL), tumor necrosis factor (TNF) and so on, and recruitment of inflammatory cells. Among these, TGF-β is known to be a major inducer of the initiation and maintenance of lung fibrosis by promoting EMT, targeting the expression of α-SMA and ECM proteins Collagen I and Fibronectin to activate the fibroblast and ECM deposition in the pathology of IPF [7–9]. Epithelial mesenchymal transition (EMT) is a process that fully differentiated epithelial cells undergo transition to the mesenchymal phenotype of fibroblasts and myofibroblasts in the organ fibrosis. As the main inducer of EMT, TGF-β/Smad signaling promotes the transformation of the epithelial cells to mesenchymal cells, which gain motility and migrate to the site of injury, where they deposit ECM and mediate the tissue remodeling, leading to fibrosis and potential organ dysfunction [10–13].

Deregulation of protein ubiquitination plays a crucial role in organ fibrosis. Ubiquitin specific peptidase 15 (USP15) is a deubiquitinase with multiple protein substrates, such as PARP, ATM, cGAS [14, 15]. It was reported that USP15 was up-regulated in hypertrophic scar tissues, and co-immunoprecipitation (Co-IP) demonstrates that USP15 interacts with and stabilize TGF-β receptor I (TBR1) and Smad2/3 to maintain TGF-β/Smad 2/3 signaling pathway in human dermal fibroblasts [16, 17]. However, whether USP15 is involved in IPF and pharmacological inhibition of USP15 could relieve IPF remain unclear.

Z86 is a β-carbolines compound with anti-tumor activity previously discovered, with Wnt/β-catenin signaling and PI3K/Akt/mTOR signaling involved in the underlying mechanisms [18, 19]. However, whether Z86 has the activity against IPF remains unclear. Herein, interestingly, we found that Z86 inhibited TGF-β induced EMT and subsequent fibroblasts differentiation and ECM deposition in A549 and HFL1 cells, and showed potential therapeutic effects in BLM caused IPF mice model. In addition, we demonstrated Z86 disrupted TGF-β/Smad2/3 signaling pathway through targeting USP15 to promote the ubiquitin and degradation of Smad2/3.

## Materials and methods

### Chemicals and reagents

Z86 was synthesized as previously reported [18], while Nintedanib was obtained from MedChemExpress (MCE). Stock solutions of the compounds in pure dimethyl sulfoxide (DMSO) were kept properly. Antibodies against Vimentin, α-SMA and GAPDH were purchased from Proteintech, antibodies for Collagen Ⅰ, Smad2/3, p-Smad2/3, N-Cadherin, E-Cadherin, were obtained from Cell Signaling Technology, while Fibronectin antibody was from Santa Cruz Biotechnology. TGF-β was purchased from Peprotech.

### Cell lines and cell culture

The human fetal lung fibroblast cell line HFL1 (#SCSP-5049) and human lung adenocarcinoma cell line A549 were purchased from Shanghai Cell Bank of Chinese Academy of Sciences. HFL1 cells and A549 cells were separately cultured in Ham’s F-12 K and RPMI-1640 medium, supplemented with 10% fetal bovine serum. All the cells were kept in a 37 °C constant temperature incubator with 5% CO_2_.

### Immunofluorescence staining

Cells were plated and allowed to adhere overnight for attachment, then the cells were incubated with the specified compounds for 48 h in the absence or presence of TGF-β (10 ng/ml). The cells were fixed in 4% formalin, permeabilized in 0.1% Triton X-100, and blocked with 3% BSA, followed by overnight incubation with primary antibodies. Then the cells were stained with corresponding fluorophore-labeled secondary antibody and DAPI ((4′,6-diamidino-2-phenylindole, DAPI), after which the cells were examined using an inverted fluorescence microscope (Nikon, Eclipse Ti2).

### Western blot analysis

Cells were incubated with compounds for 48 h with or without TGF-β. The protein extraction of cells and lung tissues were prepared as bellow. Briefly, samples were lysed in RIPA buffer supplemented with the protease and phosphatase inhibitor cocktail (Roche), and the protein was quantified with the BCA Kit (Beyotime Biotechnology). The proteins were separated with SDS-PAGE, and then transferred to the PVDF membranes. The membranes were subsequently probed with the designated primary antibodies and corresponding HRP-conjugated secondary antibody, respectively. Finally, the protein band was visualized with ECL (Thermo Scientific) using the imaging system (Tanon 5200).

### RT-qPCR

Total RNA was extracted from cells using a total RNA extraction kit according to the manufacturer's instructions (DP419, TianGen, Beijing, China). 5 × PrimeScript RT master mix (RR036A-1, Takara, Beijing, China) was used to synthesized cDNA from the RNA. RT-qPCR was performed by 2 × SYBR qPCR Master Mix (A304, GenStar, Beijing, China) using gene-specific primers obtained from Tsingke Biotechnology. Quantitative analysis was performed using the 2^−ΔΔCT^ method. The primer sequences used are listed below.

- GAPDH-F: AGAAGGCTGGGGCTCATTTG
- GAPDH-R: AGGGGCCATCCACAGTCTTC
- Vimentin-F: GACGCCATCAACACCGAGTT.
- Vimentin-R: CTTTGTCGTTGGTTAGCTGGT.
- FN1-F: CGGTGGCTGTCAGTCAAAG
- FN1-R: AAACCTCGGCTTCCTCCATAA
- COL1A2-F: GAGGGCCAAGACGAAGACATC
- COL1A2-R: CAGATCACGTCATCGCACAAC
- CDH2-F: AGCCAACCTTAACTGAGGAGT
- CDH2-R: GGCAAGTTGATTGGAGGGATG
- ACTA2-F: AAAAGACAGCTACGTGGGTGA
- ACTA2-R: GCCATGTTCTATCGGGTACTTC
- Smad2-F: CCGACACACCGAGATCCTAAC
- Smad2-R: GAGGTGGCGTTTCTGGAATATAA
- Smad3-F: TGGACGCAGGTTCTCCAAAC
- Smad3-R: CCGGCTCGCAGTAGGTAAC

### Wound-healing assay

Cell migration was assessed using a wound healing assay with A549 cells. Cells were seeded and kept attached, then the cells were starved and the wounds were made. Subsequently, the cells were incubated with Z86 in the presence or absence of TGF-β (10 ng/mL), and wound images were captured with the inverted microscope (Nikon, Eclipse Ti2). The width of migration was analyzed using ImageJ and the data were normalized to the initial wound width.

### Dual-luciferase reporter assay

Briefly, pSMAD-TA-Luc (Beyotime) plasmaid and Renilla plasmaid (Promega) were transfected into A549 cells. After 6 h, Z86 was added into cell culture media in the presence of TGF-β. After 24 h, luciferase and Renilla activities were assessed using the Dual-Glo Luciferase Assay System.

### BLM-induced IPF mice model

All experimental procedures were approved by and conducted in accordance with the guidelines of the Animal Care and Use Ethics Committee of Yunnan University (Approval No. YNU20240718). The mouse model of lung fibrosis induced by Bleomycin (BLM) is widely used and well-accepted in IPF research. Briefly, Six-week old C57BL/6 mice obtained from the Vital River were maintained under specific pathogen-free conditions and randomly allocated to three groups: a healthy control (saline), a fibrotic model (BLM), and a treatment group. Following anesthesia, mice in the latter two groups received a single intratracheal administration of 5 mg/kg BLM, with Z86 or Nintedanib therapy commencing 24 h later. The mice received daily oral gavage of the drug vehicle or Z86(1 mg/kg and 2.5 mg/kg) or Nintedanib (30 mg/kg) for 28 days.

At the conclusion of the experiment, the lungs were removed and the lung weight coefficient was computed as the lung weight by the corresponding mouse body weight. For histological evaluation, freshly collected lungs were fixed with neutral formalin, embedded in paraffin and sectioned according to the standard histological procedure. The slides were respectively subjected to hematoxylin and eosin (H&E) staining following standard protocols. For immunostaining, the lung sections were probed with antibodies against Collagen I、α-SMA、 N-cadherin、Vimentin.

### Assessment of USP15 activity using Ub-PA activity-based probes via labeling and competition assays

Following a 6-h treatment of A549 cells with Z86 at indicated concentrations (0, 5, 10, and 20 μM), cells were collected and lysed in TE buffer. After centrifugation, the supernatant was collected, diluted in probe buffer to an appropriate concentration, and mixed with 5 × loading buffer. Samples were denatured at 70 °C for 10 min and subjected to protein immunoblotting to evaluate the binding of the molecular probe to the deubiquitinase USP15, USP4 and USP8 in the presence of Z86.

### Cellular thermal shift assay (CETSA)

A549 cells were maintained in 10-cm culture plates for 12 h. After harvesting and rinsing thoroughly with PBS, the cell pellets were resuspended in a PBS buffer fortified with a comprehensive protease inhibitor cocktail, followed by lysis via three rapid freeze–thaw cycles in liquid nitrogen. The lysates were clarified by centrifugation at 12,000 × g for 20 min at 4 °C to remove cellular debris. The supernatant was partitioned into two portions: the first served as a vehicle control (treated with DMSO), while the second was exposed to Z86 (20 μM). Following a 30 min incubation at ambient temperature, both lysates were subdivided into 50 μL aliquots and heated individually at specified temperatures for 3 min, then cooled at room temperature for 3 min. Clarified supernatants were transferred to new microtubes for subsequent SDS-PAGE and Western blot analysis.

### Deubiquination assays

For the evaluation of endogenous ubiquitination, A549 cells were stimulated with TGF-β for 24 h, followed by incubation with either DMSO or Z86 (20 μM) for an additional 12 h. For the exogenous linkage-specific ubiquitination assays, cells were transiently transfected with plasmids encoding HA-tagged wild-type (WT), K48-only, or K63-only ubiquitin. Following transfection, cells were stimulated with TGF-β and co-treated with Z86 and the proteasome inhibitor MG132 (10 μM) prior to harvesting.

All cells were harvested and lysed using a lysis buffer supplemented with a protease inhibitor cocktail. For immunoprecipitation, equivalent amounts of cell lysates were incubated overnight at 4 °C with specific primary antibodies: an anti-Ub antibody (sc-53509, Santa Cruz Biotechnology, USA) was used for the endogenous assay, whereas an anti-Smad2/3 antibody was utilized for the exogenous assay. Subsequently, the immune complexes were captured by incubation with Protein A/G magnetic beads for a further 6 h. After four rigorous washes with the lysis buffer, the beads were boiled in 2 × SDS sample loading buffer for 10 min. Finally, the eluted samples were subjected to immunoblotting and the targeted proteins or specific ubiquitin linkages were detected using the specified antibodies (e.g., anti-HA).

### Molecular docking

Molecular docking studies between ligand molecules and receptors were performed using the Ligand Docking module implemented in Maestro 2025.1 (Schrödinger, LLC). The LigPrep module was employed to preprocess the compound structures imported in mol2 format. Parameters were configured to automatically add hydrogen atoms, optimize molecular geometry, and generate diverse conformational states for each ligand. Energy minimization was carried out using the OPLS4 force field. The resulting ligand structures were rigorously validated to ensure structural integrity and appropriate charge states, thereby fulfilling the prerequisites for subsequent docking analyses. For protein preparation, individual receptor structures were imported and processed using the Protein Preparation Wizard. The simulation pH was set to 7.4 in the global settings of the workflow, with all other parameters maintained at their default values. Prepared protein structures were carefully inspected and saved for subsequent docking steps. A receptor grid was generated with a scaling factor of 0.8 using the Receptor Grid Generation panel. Preprocessed ligands and receptor structures were loaded into the Ligand Docking workflow in compressed format. Docking simulations were performed in standard precision (SP) mode employing flexible ligand sampling and a van der Waals scaling factor of 0.8 applied to both receptor and ligand atoms.

### Statistical analysis

Data are expressed as mean ± SD. Statistical comparisons were made using Student's t-test with GraphPad Prism software (version 8). Values of #p < 0.05, ##p < 0.01, and ###p < 0.001 were considered significant between BLM alone vs control groups or TGF-β vs control groups. Values of *p < 0.05, **p < 0.01, ***p < 0.001 were considered significant between BLM or TGF-β alone vs other treatment groups.

## Results

### Z86 ameliorated BLM-induced pulmonary fibrosis in mice

**Fig. 1:**
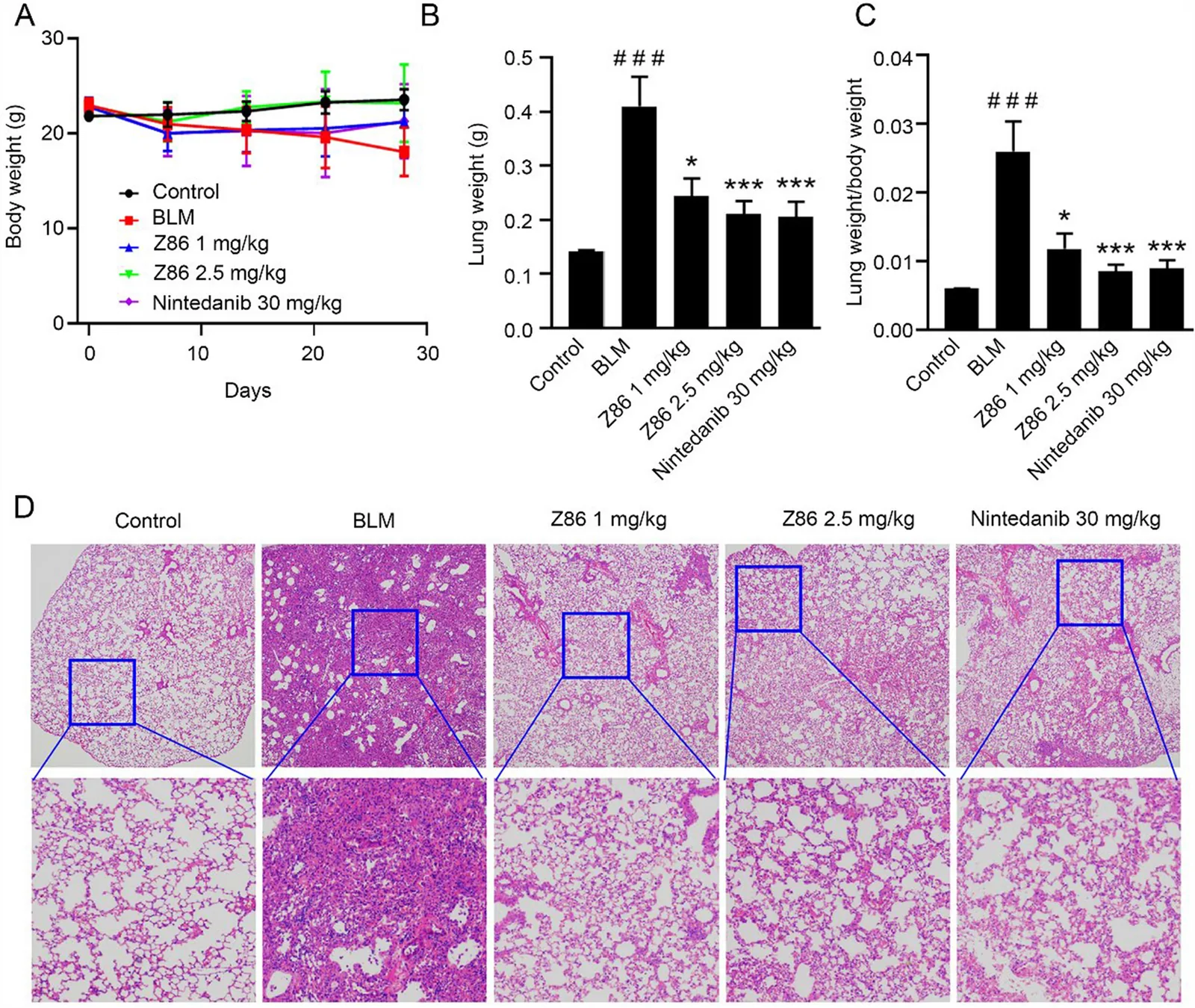
Z86 ameliorated BLM-induced weight loss and lung injury in mice. C57BL/6 mice were injected intratracheally with 0.9% NaCl solution as control or BLM, then the BLM-challenged mice were administered orally with Z86 (1 mg/kg, 2.5 mg/kg) or Nintedanib (30 mg/kg) daily for 4 weeks. **A-C** The mice weight was documented, and the lung weight and the ratio of lung weight/body weight was calculated. **D** HE staining was performed to demonstrate the pathological changes in the lungs of mice

To verify whether Z86 could protect pulmonary from fibrosis in vivo, the BLM-induced pulmonary fibrosis model [21] was established. Z86 was administered orally once daily for a total period of 28 days, with the clinically utilized Nintedanib, an intracellular inhibitor that targets multiple tyrosine kinases including the VEGF, FGF, and PDGF receptors, as control. BLM administration led to body loss over the experimental period compared to the control group. As expected, the body weight loss was alleviated in the mice received Z86 (1 mg/kg) or Nintedanib compared to the BLM group. The mice of 2.5 mg/kg Z86 treated group maintained their body weights compared with the control group (Fig. 1A). Moreover, BLM led to increased lung weights and ratio of lung weight/body weight, and the increase was strikingly reduced in mice treated with Z86 to the level similar to the Nintedanib group (Fig. 1B, C). The results above suggested Z86 might relieve BLM-induced pulmonary fibrosis in vivo. HE staining assay was further performed to determine the histological changes in the lung tissues. As shown in Fig. 1D, the normal alveolar framework revealed in control group was severely destroyed by BLM, while Z86 or Nintedanib administration resulted in restoration of almost intact alveolar structure, suggesting Z86 exhibited potential anti-fibrosis activity in mice.

### Z86 attenuated EMT, myofibroblast accumulation and ECM deposition in BLM-induced fibrotic lungs

**Fig. 2:**
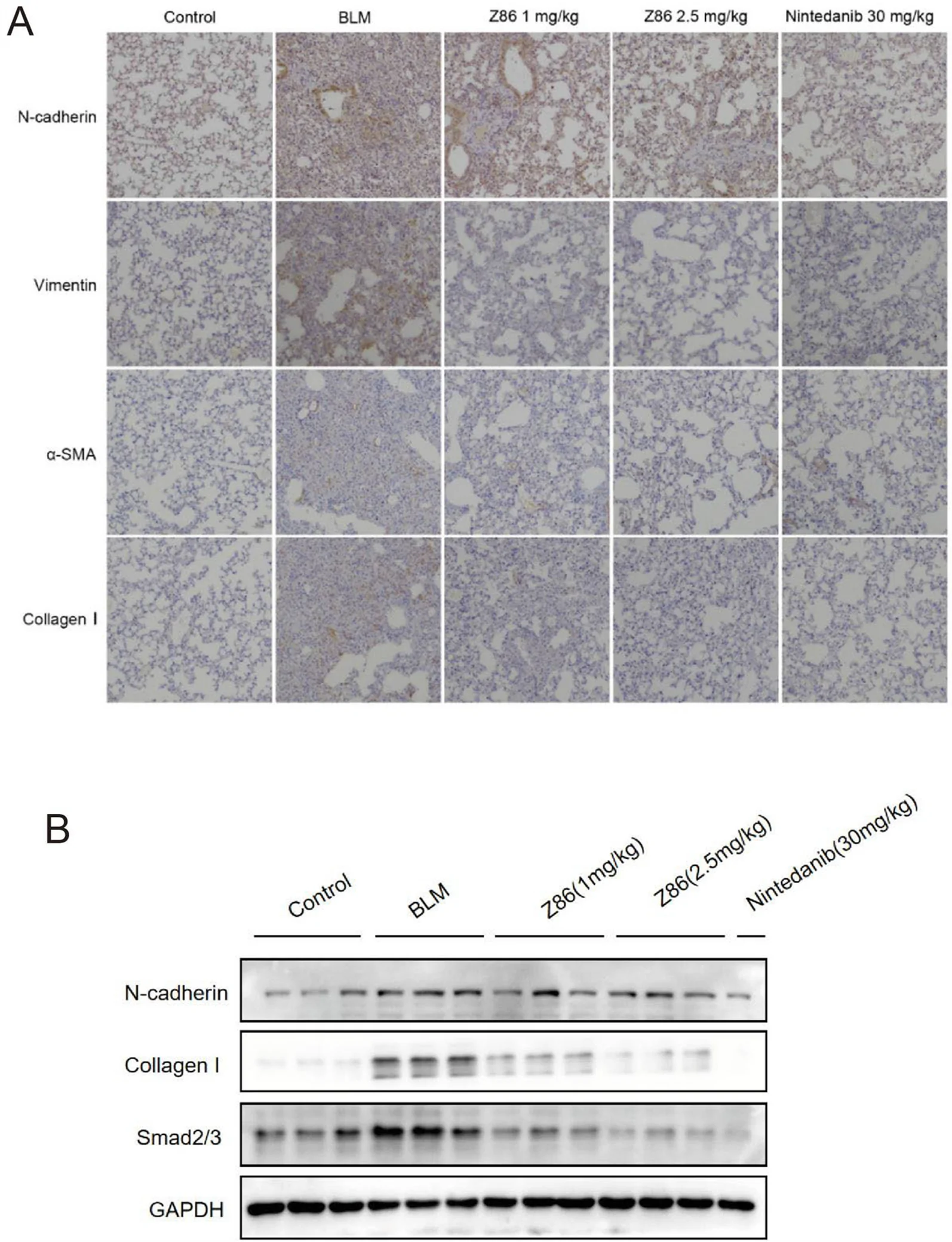
Z86 attenuated EMT, myofibroblast accumulation and ECM deposition in BLM-induced fibrotic lungs. **A** Immunohistochemical staining of pro-fibrotic proteins N-cadherin, Vimentin, α-SMA and Collagen I in lung tissues. Scale bar: 100 μm. **B** Western blot analysis of N-cadherin, Collagen I and Smad2/3, protein levels in lung tissues

To further quantify the anti-fibrosis effect and explore the underlying mechanism of Z86 in vivo, the pro-fibrotic proteins, including the EMT molecules N-cadherin, Vimentin, the myofibroblast activation characterized by ɑ-SMA, as well as the ECM Collagen I deposition were detected in lung sections with the immunohistochemical analysis, respectively. As shown in Fig. 2A, compared to the control group the levels of EMT proteins N-cadherin and Vimentin, myofibroblast activation marked by ɑ-SMA in BLM group were significantly increased, along with increased Collagen I deposition. While the mice received Z86 or Nintedanib treatment revealed less and weaker positive staining, suggesting Z86 suppressed BLM induced lung fibrosis in vivo. To further confirm the mechanism of action, the protein levels of the fibrosis-associated proteins N-cadherin, Collagen I, as well as Smad2/3 in the lung samples were detected with the Western blot analysis. As shown, Z86 significantly decreased the levels N-cadherin and Collagen I in lung tissues, which is consistent with the results of IHC analysis (Fig. 2B). More importantly, Smad2/3, the marker of mechanism showed decreased in the Western blot assay, confirming the blockade of the TGF-β/Smad signaling pathway in vivo.

### Z86 attenuated TGF-β induced EMT in A549 and HFL1 cells

**Fig. 3:**
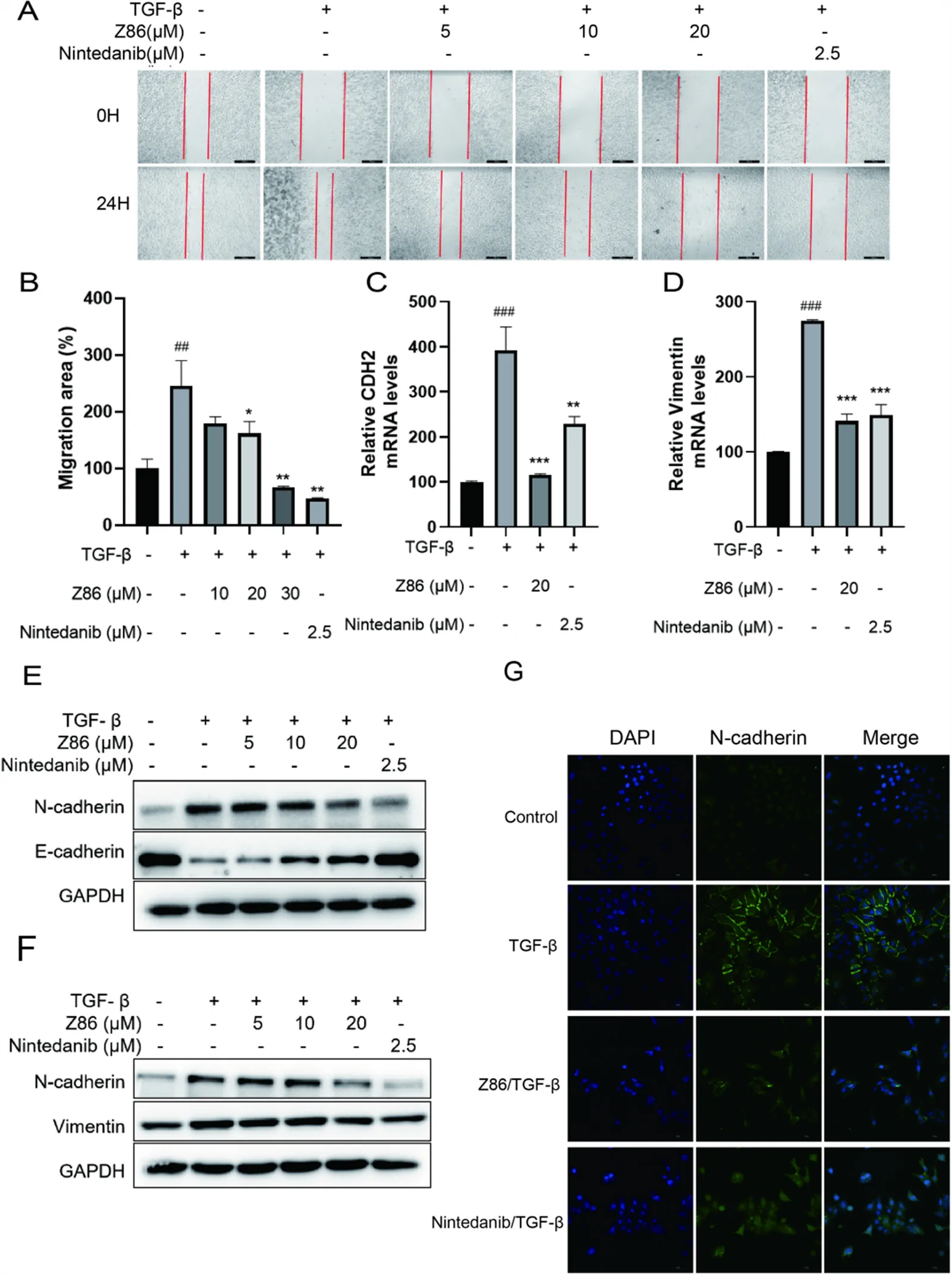
Z86 attenuated TGF-β induced EMT in A549 and HFL1 cells. **A** Z86 inhibits TGF-β1-induced A549 cells migration as analyzed by wound healing assay, Scale bar: 100 μm. **B** Quantitative analysis of the migration rates in (**A**). **C-D** The relative mRNA expression of CDH2 and Vimentin in A549 cells was determined by qPCR. **E** The protein level of N-cadherin and E-cadherin in A549 cells was assessed by WB. **F** The protein level of N-cadherin and Vimentin in HFL1 cells was assessed by WB. **G** Immunofluorescence determined the expression of N-cadherin in A549 cells. (bars = 10 μm). Data was analyzed as mean ± SD. *p < 0.05, **p < 0.01, and ***p < 0.001 vs. the model group; #p < 0.05, ##p < 0.01, and ###p < 0.001 vs. the control group

EMT is a crucial stage in the myofibroblast activation. Following the damage of lung epithelial cell, EMT is activated and induces an abnormal myofibroblast activation and subsequent excessive deposition of ECM and remodeling of the lung interstitium [19]. To further clarify the activity and mechanism of Z86 in IPF, we detected the effect of Z86 on TGF-β1-induced EMT in cells. A549 cells are human lung cancer cells that retain the characteristics of alveolar epithelial-type II cells, and human fetal lung fibroblast-1 cell line (HFL1) is a primary human lung fibroblast cell line. Both of these cells are the primary origins of myofibroblasts and produce ECM in response to TGF-β1 induction and are widely used as in vitro cell model in the study of IPF. Firstly, wound healing assay was used to detect the migration of A549 cells. As shown in Fig. 3 A, B, TGF-β1 enhanced the migration of A549 cells due to remodeled cytoskeletal structure, while Z86 impeded the abnormal migration induced by TGF-β1. Moreover, the expression of EMT markers CDH2 (encoding N-cadherin) and Vimentin were detected by qPCR and western blotting at mRNA and protein levels in A549 and HFL1 cells (Fig. 3C–F). The results indicated the treatment of Z86 increased the expression of E-cadherin and decreased the expression of N-cadherin and Vimentin compared with the model group. In addition, the level of N-cadherin was also downregulated in the immunofluorescence assay performed in A549 cells (Fig. 3G). In summary, Z86 effectively suppressed TGF-β1 induced EMT process to alleviate IPF.

### Z86 diminished TGF-β induced fibroblast activation and ECM production

TGF-β induced EMT promotes fibroblasts activation and differentiation into myofibroblasts, resulting in collagen deposition and increased extracellular matrix [18]. Thus, the activation of fibroblasts and ECM deposition were detected in Z86 treated cells.

**Fig.4:**
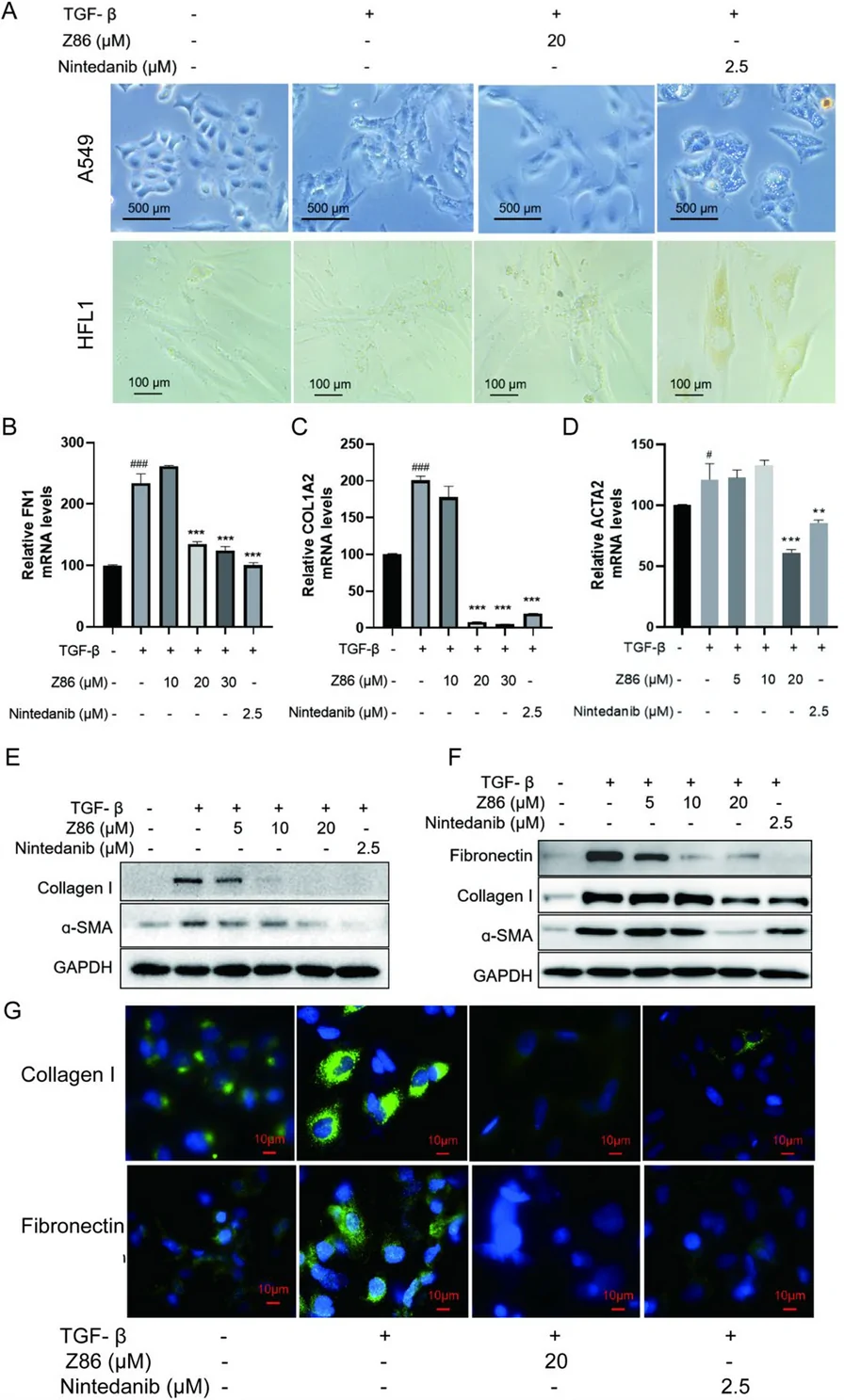
Z86 alleviated TGF-β1 induced ECM production in A549 and HFL1 cells. **A** The effect of Z86 on A549 cells (up, bars = 500 μm) and HFL1 cells (down, bars = 100 μm) morphology change. **B–D** The relative mRNA expression of FN1, COL1A2 and ACTA2 in A549 cells was determined by qPCR. **E** The protein level of Collagen I and α-SMA in A549 cells was assessed by WB. **F** The protein level of Fibronectin, Collagen I and α-SMA in HFL1 cells was measured by WB. **G** Immunofluorescence determined the expression of Collagen Ⅰ and Fibronectin in A549 cells (bars = 10 μm). Data was analyzed as mean ± SD. *p < 0.05, **p < 0.01, and ***p < 0.001 vs. the model group; #p < 0.05, ##p < 0.01, and ###p < 0.001 vs. the control group

Firstly, A549 cells and HFL1 cells were subjected to cell morphology analysis. As shown in Fig. 4A, A549 cells without any stimulation showed cobblestone-like epithelial morphology, while TGF-β1 contributed to the transition of fibroblast-like morphology to myofibroblas-like cell morphology and induced cell phenotypic changes into spindle-like morphology. However, treatment of Z86 (20 μM), with Nintedanib (2.5 μM) as control, restored the TGF-β induced morphological changes in both the cells, indicating Z86 suppressed the fibroblast activation.

Further RT-qPCR, Western blot and immunofluorescence assays were used to detect the expression of α-SMA and ECM related proteins (Collagen I, Fibronectin) in mRNA and protein levels to confirm the inhibition of Z86 in fibroblast activation and ECM deposition. In the qPCR assay, TGF-β1 elevated the transcription of ACTA2 (encoding α-SMA), FN1 (encoding Fibronectin), and COL1A2 (encoding Collagen I), and the elevated transcription was alleviated in A549 cells treated with Z86 (Fig. 4 B-D). Moreover, Western blot analysis illustrated that the protein expression of α-SMA, Fibronectin and Collagen I were significantly decreased in A549 and HFL1 cells exposed to Z86 compared with the TGF-β1 group (Fig. 4 E–F). Consistently, the reduced ECM deposition was further confirmed by the fluorescence intensity of Collagen I and Fibronectin in Z86 treated A549 cells with immunofluorescence assay (Fig. 4 G). These results indicate that Z86 was capable of inhibiting TGF-β1 induced fibroblast activation and ECM remodeling in lung-derived cells.

### Z86 attenuated TGF-β/Smad2/3 pathway and decreased Smad 2/3 protein level

**Fig. 5:**
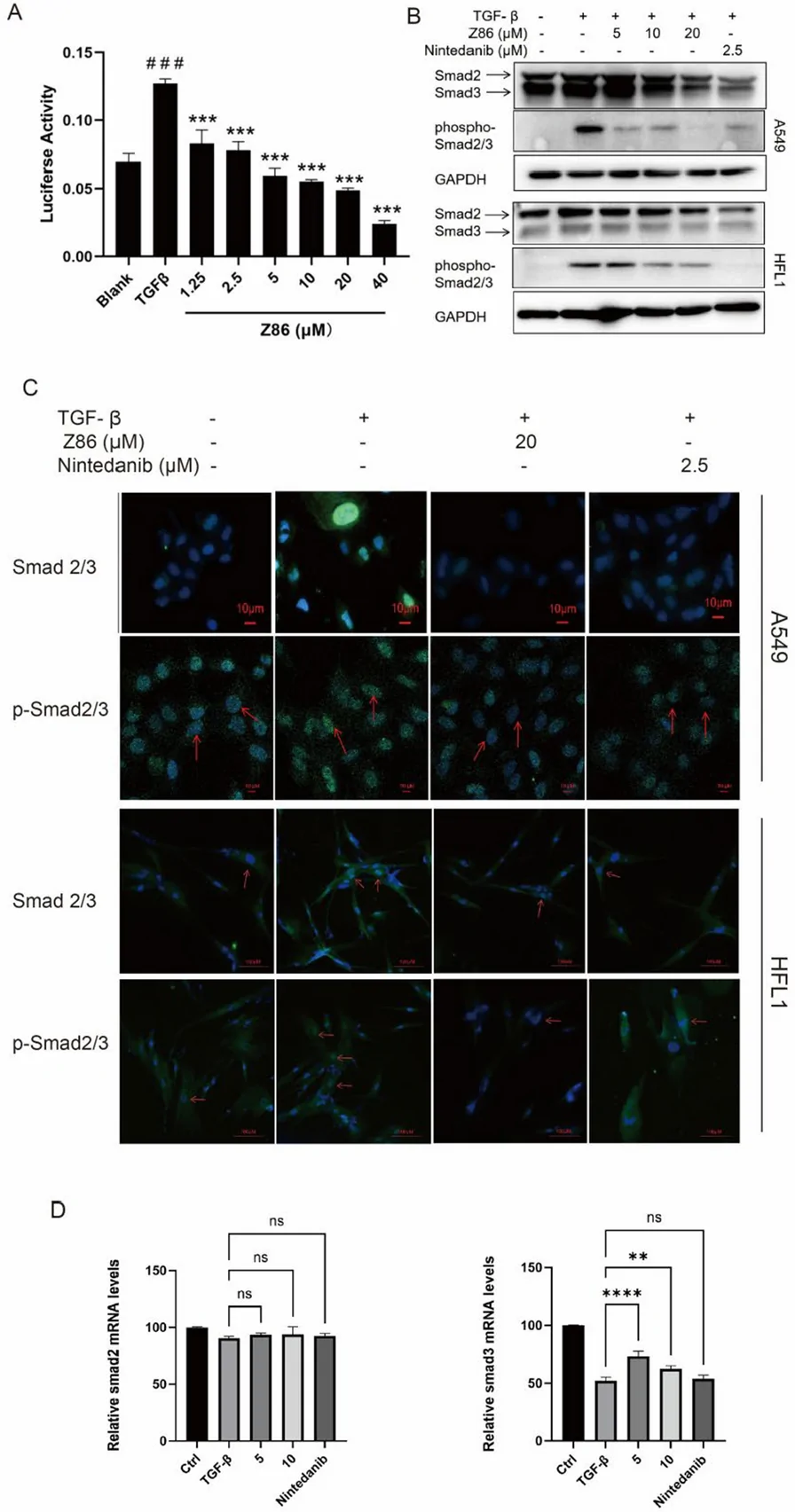
Z86 attenuated TGF-β/Smad2/3 pathway in IPF cells. **A** The reporter gene activity of TGF-β/Smad2/3 pathway in A549 cells treated with Z86. **B** The protein levels of phospho-Smad2/3 and total Smad2/3 in A549 cells and HFL1 cells were assessed by western bolt. **C** Immunofluorescence determined the distribution of Smad2/3 in A549 and HFL1 cells (bars = 10 μm). **D** The relative mRNA expression of Smad2 and Smad3 in A549 cells was determined by qPCR. Data was analyzed as mean ± SD. *p < 0.05, **p < 0.01, and ***p < 0.001 vs. the model group; #p < 0.05, ##p < 0.01, and ###p < 0.001 vs. the control group

It is widely accepted that TGF-β1 promotes the progression of pulmonary fibrosis through the activation of Smad 2/3 to promote EMT, the transformation of fibroblasts into myofibroblasts, resulting in the accumulation of ECM [23]. Therefore, we concentrated on the inhibitory activity of Z86 on the TGF-β/Smad2/3 pathway. Firstly, the TGF-β/Smad2/3 signaling was detected with dual-luciferase reporter assay. TGF-β induced dramatic activation of TGF-β/Smad2/3 pathway, while Z86 effectively reduced the aberrant TGF-β/Smad2/3 activation induced by TGF-β1 (Fig. 5A). Besides, the level of Smad2/3 and phosphorylated Smad2/3 by western blot analysis and immunofluorescence assay were also detected. As shown in Fig. 5B, C the phosphorylated Smad 2/3 induced by TGF-β was alleviated by Z86 as well as Nintedanib. Of note, the total Smad2/3 level was reduced as well. To further investigate the mechanism of Smad2/3 downregulation, the mRNA levels of Smad2/3 were detected with Real-time PCR assay, while no obvious downregulation of Smad2/3 mRNA levels were observed (Fig. 5D), indicating Z86 downregulates Smad2/3 at the post-translational level.

### Z86 targets and modulates the USP15-Smad2/3 signaling pathway

**Fig. 6:**
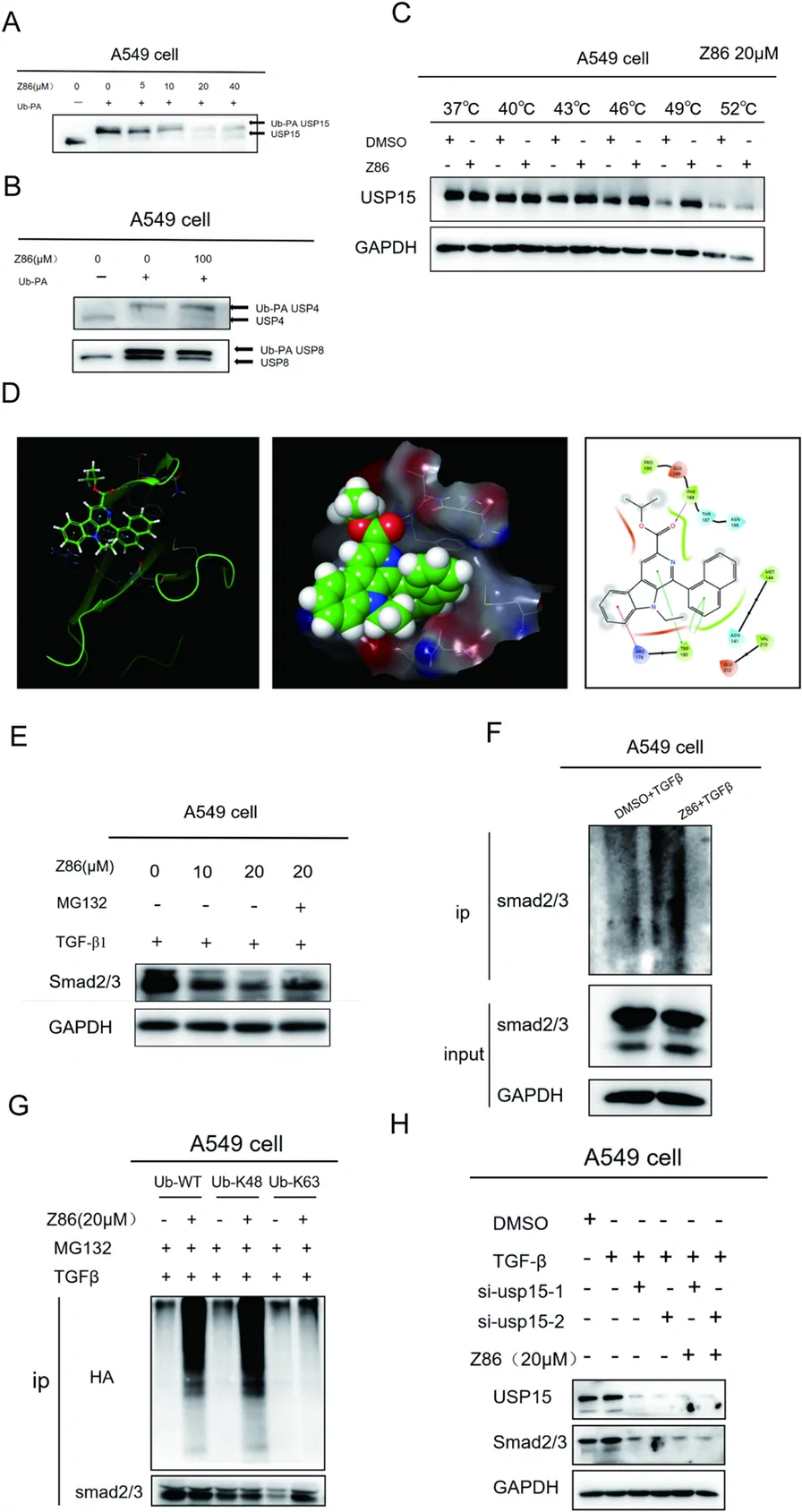
Z86 targets USP15 to promote the ubiquitination and degradation of Smad2/3. **A** Cells were treated with the indicated concentrations of Z86 for 6 h, and lysates were incubated with the active-site probe Ub-PA. **B** Evaluation of DUB selectivity. TGF-β-induced A549 cells were treated with Z86, and lysates were subjected to the Ub-PA assay to assess the activity of USP8 and USP4. **C** Cellular thermal shift assay (CETSA) in A549 cells treated with DMSO or 20 μM Z86 for 30 min. **D** Molecular docking analysis of Z86 binding to USP15. Overlay of the top scoring docking poses of Z86, surface representation of the USP15 catalytic domain with Z86 (stick model) docked in the binding pocket, and a close-up 2D schematic of predicted interactions. **E** Effect of Z86 and the proteasome inhibitor MG132 (10 μM, 6 h) on Smad2/3 protein levels in TGF-β-induced A549 cells. **F** Immunoprecipitation (IP) of Smad2/3 followed by immunoblotting (IB) for ubiquitin (Ub) in TGF-β-induced A549 cells treated with Z86. **G** Assessment of K48-linked and K63-linked polyubiquitination of Smad2/3 following Z86 treatment. **H** Immunoblot analysis of Smad2/3 protein levels in control and USP15-knockdown A549 cells treated with or without Z86

Potential target of Z86 in TGF-β/Smad signaling suppression, especially in the Smad2/3 degradation was further explored. USP15 has been previously implicated in stabilizing Smad2/3 and positively regulating the TGF-β pathway in the context of glioma and dermal fibrosis [16, 17]. A Ub-PA assay was performed to evaluate the potential inhibition of USP15 by Z86 (Fig. 6A), and the results revealed that Z86 induced a profound suppression of USP15 activity. To confirm the DUB selectivity of Z86, Ub-PA assay based deubiquitinase activity of Z86 was conducted with USP8 and USP4 (the closest isoform of USP15), while no dominant USP8 or USP4 inhibition was observed ( Fig. 6B), indicating that Z86 exerts a relatively selective inhibition on USP15. Furthermore, the cellular thermal shift assay (CETSA) demonstrated that Z86 enhanced the thermal stability of USP15 (Fig. 6C), confirming the interaction between USP15 and Z86. To elucidate the structural basis of the interaction between Z86 and USP15, molecular docking studies were performed using the Schrödinger software suite. The results predict that Z86 binds snugly within the catalytic cleft of USP15, forming specific hydrogen bonds and hydrophobic interactions with key residues (Fig. 6D).

We therefore investigated whether Z86 promotes the ubiquitination of Smad2/3 by targeting USP15. The ubiquitination assay showed that treatment of TGF-β-induced A549 cells with Z86 resulted in an increase in ubiquitinated Smad2/3 and a subsequent reduction in total Smad2/3 levels (Fig. 6F). Furthermore, the proteasome inhibitor MG132 (10 μM, 6 h) prevented the Z86-induced decrease in Smad2/3 (Fig. 6E), suggesting that this degradation is dependent on the ubiquitin–proteasome system. To specify the ubiquitin chain linkage involved in this process, we assessed K48-linked and K63-linked polyubiquitin chains were applied. Z86 treatment significantly enhanced the K48-linked polyubiquitination of Smad2/3, the canonical signal for proteasomal degradation, whereas no significant change was observed in K63-linked ubiquitination (Fig. 6G). To determine whether Z86 induced Smad2/3 degradation is USP15-dependent, we evaluated Smad2/3 levels in USP15-knockdown cells. As shown in Fig. 6H, USP15 knockdown effectively reduced Smad2/3 protein levels, and Z86 treatment failed to further alter the amount of Smad2/3 in these cells, indicating that Z86-induced Smad2/3 degradation is largely USP15-dependent. Collectively, these data demonstrate that Z86 exerts its anti-fibrotic effects by inhibiting USP15, thereby promoting the K48-linked polyubiquitination and subsequent proteasomal degradation of Smad2/3 to interrupt aberrant TGF-β/Smad signaling.

## Discussion

IPF is a life-threatening lung disease with high morality and morbidity and mortality, which highlight the great need for novel potential agents. IPF is characterized by lung function disorder and subsequent failure due to excessive accumulation of ECM [20]. The myofibroblast cells are the critical ECM-secreting cells and responsible for ECM accumulation and progression of fibrosis [21], and originate from epithelium suffered EMT, local fibroblasts differentiation, mesenchymal endothelia, even macrophages transformation under inflammatory stimulation [22]. Accordingly, pharmacological agents interfering with the pro-fibrotic myofibroblasts including the EMT of epithelium, fibroblasts transition, as well as survival of myofibroblasts are effective strategies in antifibrotic therapy [23]. Herein, we discovered the anti-fibrotic potential of Z86 in BLM induced pulmonary fibrosis in vivo. In specific, Z86 restored almost intact alveolar structure as well as weight loss and lung weight increase in BLM-induced pulmonary fibrotic mice. Furthermore, the EMT markers Vimentin and N-cadherin, the fibroblast activation marker α-SMA and ECM deposition Collagen I were all alleviated in the immunohistochemical analysis, which were also confirmed in TGF-β treated lung alveolar epithelial A549 cells and primary lung fibroblast HFL1 cells. Taken together, our founding indicated the potential antifibrotic efficacy of Z86 in pulmonary fibrosis.

Multiple signaling pathways including TGF-β/Smad, WNT/β-catenin, Notch work together to regulate pulmonary fibroblast, with TGF-β/Smad signaling as the well identified cascade involved in IPF. TGF-β, mainly produced by M2 macrophages, is a critical pro-fibrotic factor in IPF [24]. Binding of TGF-β to cognate receptors induces phosphorylation and nucleus translocation of Smad2/3 and formation of transcriptional Smad complex, leading gene transcription of EMT transition and ECM molecules collagen, fibronectin [12]. Moreover, deficiency of Smad3 contributed to bleomycin-induced pulmonary fibrosis suppression [25], while the FDA approved anti-fibrosis drugs Nintedanib and Pirfenidone also exhibited TGF-β/Smad cascade inhibition [21, 26], indicating TGF-β/Smad signaling is the potential therapeutic target in IPF. Although we previously reported the Wnt/β-catenin signaling pathways inhibitory activity of Z86, in this study, we found Z86 demonstrated potent TGF-β/Smad cascade suppression in the dual-luciferase assay, and the phosphorylation of Smad2/3 induced by TGF-β was also strikingly rescued in the subsequent western blot analysis. In spite of the involvement of Wntβ-catenin in IPF, Z86 showed much stronger inhibitory activity on TGF-β/Smad cascade with relative weaker Wnt/β-catenin (data not shown), indicating Z86 preferentially inhibited TGF-β/Smad cascade in the context of IPF.

Emerging evidence highlights the pivotal role of ubiquitin modification in the IPF pathogenesis especially in the TGF-β/Smad signaling pathway that is tightly correlated with the development of IPF. Although ubiquitin ligases such as TRIM47, Smurf2 were reported to be involved in IPF by interacting with the TGF-β/Smad signaling components [27], the involvement of DUBs has been rarely reported. USP15 binds to Smad2/3 and is recruited to the TGF-β receptor complex, where it deubiquitinates and stabilizes TBR-I and Smad 2/3, thereby promoting sustained activation of TGF-β signaling in human dermal fibroblasts. Moreover, USP15 has been reported to promote the differentiation of fibroblasts into myofibroblasts, facilitating extracellular matrix remodeling during wound healing in skin wound repair. USP7 inhibitor P22077 inhibited BLM induced IPF by reducing the expression of ECM [28]. In this study, we find Z86 as an inhibitor of USP15, suppressed the occurrence of IPF through Smad2/3 degradation and TGF-β/Smad signaling inhibition, suggesting USP15 is tightly involved in the regulation of pulmonary fibrosis and represents a potential therapeutic target worthy of further investigation.

## Data Availability

Data will be available on request.

## Abbreviations

TGF-β: Transforming growth factor β
EMT: Epithelial-mesenchymal transition
ECM: Extracellular matrix
IPF: Idiopathic pulmonary fibrosis
